# Common features in diverse insect clocks

**DOI:** 10.1186/s40851-014-0003-y

**Published:** 2015-02-20

**Authors:** Hideharu Numata, Yosuke Miyazaki, Tomoko Ikeno

**Affiliations:** Graduate School of Science, Kyoto University, Kyoto, 606-8502 Japan; Graduate School of Education, Ashiya University, Ashiya, 659-8511 Japan; Department of Psychology, Michigan State University, East Lansing, MI 48824 USA

**Keywords:** Anatomical location, Celestial navigation, Circadian, Circalunar, Circannual, Circasemilunar, Circatidal, Clock gene, Phase response curve, Photoperiodism

## Abstract

This review describes common features among diverse biological clocks in insects, including circadian, circatidal, circalunar/circasemilunar, and circannual clocks. These clocks control various behaviors, physiological functions, and developmental events, enabling adaptation to periodic environmental changes. Circadian clocks also function in time-compensation for celestial navigation and in the measurement of day or night length for photoperiodism. Phase response curves for such clocks reported thus far exhibit close similarities; specifically, the circannual clock in *Anthrenus verbasci* shows striking similarity to circadian clocks in its phase response. It is suggested that diverse biological clocks share physiological properties in their phase responses irrespective of period length. Molecular and physiological mechanisms are best understood for the optic-lobe and mid-brain circadian clocks, although there is no direct evidence that these clocks are involved in rhythmic phenomena other than circadian rhythms in daily events. Circadian clocks have also been localized in peripheral tissues, and research on their role in various rhythmic phenomena has been started. Although clock genes have been identified as controllers of circadian rhythms in daily events, some of these genes have also been shown to be involved in photoperiodism and possibly in time-compensated celestial navigation. In contrast, there is no experimental evidence indicating that any known clock gene is involved in biological clocks other than circadian clocks.

## Introduction

Biological clocks are an essential mechanism by which organisms adapt to cyclic environmental changes. Although in the 1960s and 1970s there were debates on whether biological rhythms have an endogenous nature [1], currently the existence of biological clocks is undisputed. In studies on biological clocks, insects have been shown to play many important roles.

The circadian clock has a free-running period of approximately 24 h and is entrained to exactly 24 h by daily environmental cues (Zeitgebers) such as light and temperature. This clock controls daily rhythmicity in behavior (e.g., locomotion, feeding, mating, and oviposition), physiological functions, and developmental events such as hatching, pupation, and eclosion in insects [2]. Individual insects have active and inactive phases throughout the day, providing easily understandable examples of how behavioral or physiological rhythms are produced by circadian clocks. In contrast, developmental events such as hatching, pupation, and eclosion occur only once in the life cycle of an individual insect, and therefore it is less easy to understand how a clock controls such an event. Nevertheless, using the concept of the ‘‘gate’’, Pittendrigh [3] explained that a rhythm in such an event is also produced by a circadian clock. The circadian clock entrained to a day opens the gate once a day, and the developmental event is allowed to occur only when the gate opens. In this review, we collectively refer to the circadian clock for behavioral, physiological, and developmental rhythms as the clock for daily rhythmic events.

Another clock-related phenomenon was shown in the honey-bee, *Apis mellifera* [4]. Workers of this species inform other workers in the same hive about the direction and distance of a food resource by a “waggle dance”. The direction of the waggle dance is closely correlated with the angle formed between the resource and the sun. This orientation mechanism is called a “solar compass” or “sun compass”. The monarch butterfly, *Danaus plexippus*, uses a similar mechanism in its long-distance migration [5]. Some insects use other celestial objects for orientation. A nocturnal dung beetle, *Scarabaeus zambesianus*, orients itself with polarized moonlight, and another species, *Scarabaeus satyrus*, uses the Milky Way to orient itself on moonless nights [6,7]. These mechanisms, in addition to the solar compass, are called “celestial navigation”. Celestial navigation generally needs to be compensated by time, and the existence of a biological clock should be a prerequisite for this mechanism.

Photoperiodism, a response to the length of the light or dark period in a day, has been identified in many insects, and has been shown to regulate diapause, seasonal morphs, growth rate, migration strategy, and a variety of associated physiological states [2,8,9]. Bünning [10] suggested that an internal clock may be involved in the measurement of day or night length. This hypothesis, later called “Bünning’s hypothesis”, was not accepted in its original form, but the general concept that a circadian clock is involved in photoperiodism has been accepted [11-13].

The physiological mechanisms for these diverse clock-related phenomena (i.e., circadian rhythms in daily events, time-compensated celestial navigation, and photoperiodism) involve the circadian clock entrained to a day under natural conditions. In contrast, there are also endogenous rhythms with a period considerably different from a 24 h day in organisms including insects. These rhythms have free-running periods close to the tidal (12.4 h), semilunar (14.8 days), lunar (29.5 days), or annual (365 days) period [2]. The varied carpet beetle, *Anthrenus verbasci*, shows a circannual rhythm in pupation, and with this rhythm pupation occurs only in spring [14,15]. A marine midge, *Clunio marinus*, lives in the lowest parts of the intertidal zone. Larvae of this species have a circasemilunar or circalunar rhythm in pupation and, as a result, adults emerge in spring tides [16]. The mangrove cricket, *Apteronemobius asahinai*, has a circatidal locomotor rhythm; it forages on the floor of mangrove forests during low tide and rests during high tide [17]. There are hypotheses that a circadian clock produces a circannual, circasemilunar, or circatidal rhythm [18-20]. Moreover, circa(semi)lunar rhythms can be explained by beat reactions of circadian and circatidal clocks [21]. Whether a circadian clock is involved in the generation of these non-circadian rhythms has been examined in the above three species, but no experimental evidence supporting such involvement has been found [22-28].

Here we emphasize the significance of three major findings in the history of studies on biological clocks. One is construction of a phase response curve (PRC). A PRC is a plot of phase shifts as a function of the oscillator phase at which a stimulus is given. In circadian rhythms, PRCs are usually constructed by giving a light pulse on a free-running rhythm in constant darkness [29-31]. In insects, a PRC in this form was first identified by Pittendrigh and Bruce [32] in the fruit fly *Drosophila pseudoobscura*, and such PRCs have been reported in various species [2]. PRCs not only explain the mode of entrainment but also prove the existence of an oscillator with a period of approximately a day. The second major finding is the discovery of an anatomical location of a clock. Nishiitsutsuji-Uwo and Pittendrigh [33] first anatomically localized the circadian clock governing the locomotor activity rhythm in the optic lobes of the Madeira cockroach, *Rhyparobia* (formerly *Leucophaea*) *maderae*, by performing central nervous system surgery, and later this was verified by *in vitro* experiments in *R. maderae* and the two-spotted cricket, *Gryllus bimaculatus* [34,35]. Thus, the biological clock was shown to be a physical mechanism. The third important finding is the discovery of the first clock gene, *period* (*per*), for the genetic basis of the biological clock in the fruit fly *Drosophila melanogaster*, by Konopka and Benzer [36] in 1971. Since then, understanding of the molecular mechanism of circadian clocks has advanced considerably [2,37,38]. However, the studies have mostly concentrated on the circadian clock controlling daily rhythmic events.

In this review, we survey the common features in various clocks in insects with special reference to the PRC, anatomical location of the clock, and the role of clock genes. All known clock genes were first discovered as genes that control circadian rhythms, and we refer to them simply as “clock genes” without the qualifier “circadian” in this review. Because comprehensive reviews on the mechanisms of the circadian clocks controlling daily rhythmic events have been published in recent years [37-40], we briefly survey such circadian clocks, and devote most of the review to other clocks.

### Phase response curve

#### Circadian clock for daily rhythmic events

In early studies, PRCs of the circadian rhythm in insects were intensively examined in the eclosion rhythm of *D. pseudoobscura* by Pittendrigh and his co-workers. General features of the phase response of circadian rhythms were established by those studies [29,32,41]. PRCs reveal that the direction and magnitude of a phase shift depend on the phase at which a stimulus is given. Under constant conditions, subjective day (the phase corresponding to daytime) and subjective night (the phase corresponding to nighttime) alternate approximately every 12 h in the circadian cycle. A light pulse applied in early subjective night of the circadian cycle generally delays a phase, whereas a light pulse in late subjective night advances a phase. A pulse applied in subjective day has little or no effect on the phase. Under natural conditions, therefore, light advances the rhythm in the morning or delays it in the evening, so that entrainment can be accomplished. The characteristics of phase-dependent phase shifts have been shown to be similar in circadian rhythms of other organisms examined. PRCs produced with single light pulses not only successfully explain the mechanism of entrainment by light–dark cycles, but can also be used as probes to detect the phase, period, and amplitude of circadian clocks [2,29-31].

**Figure 1:**
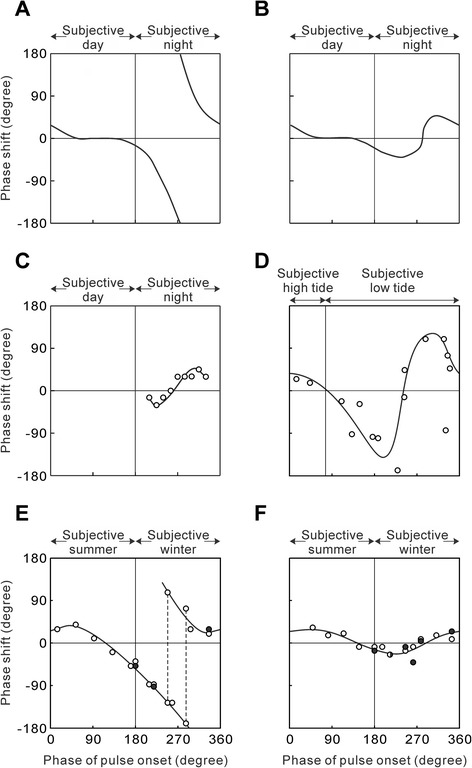
**Comparison of phase response curves (PRCs) in diverse insect clocks. (A)** Type 0 PRC in circadian clocks in response to light pulses. **(B)** Type 1 PRC in circadian clocks in response to light pulses. **(C)** PRC in the circadian clock for photoperiodism in *Sarcophaga argyrostoma* in response to 15-min light pulses (redrawn from [43]). **(D)** PRC in the circatidal clock of *Apteronemobius asahinai* in response to periodic inundations (0.5-h inundation pulses provided four times at intervals of 12.4 h) (reproduced from [45] with kind permission from Elsevier). **(E)** PRC in the circannual clock of *Anthrenus verbasci* in response to 4-week long-day pulses (reproduced from [46] with kind permission from Springer Science+Business Media). **(F)** PRC in the circannual clock of *A. verbasci* in response to 2-week long-day pulses (reproduced from [47] with kind permission from Springer Science+Business Media). The periods of clocks are shown in terms of angle degrees (0–360°). In circannual PRCs **(E, F)**, open and closed circles represent the phase shifts in the first and second pupation group after pulse perturbation, respectively, and broken lines in **(E)** show the split into advanced and delayed groups.

Winfree [42] systematically examined responses to single blue-light pulses at an intensity of 0.1 W/m^2^, and of different durations at different phases in the eclosion rhythm of *D. pseudoobscura*. He found that the pattern of resetting of the rhythm can be divided into two types: Type 0 in the case of pulse lengths > 50 s, and Type 1 in the case of pulse lengths < 50 s. Type 0 PRCs display large phase shifts and have high amplitude and a break point at the transition between delays and advances (Figure 1A). Type 1 PRCs display small phase shifts and have low amplitude and a continuous transition between delays and advances (Figure 1B) [2,30,31]. Phase resetting depends on the strength of a pulse, i.e., intensity, duration, or both, in circadian rhythms of other species as well [2]. In the eclosion rhythm of the flesh fly *Sarcophaga argyrostoma*, for example, PRCs are Type 0 with white-light pulses (2.4 W/m^2^) longer than 4 h, and Type 1 with pulses shorter than 4 h [43,44].

Type 1, but not Type 0, PRCs can be obtained if a clock is a one-dimensional oscillator. Biological examples of such oscillators are developmental sequences, cell cycles, and mammalian estrous cycles, in all of which the phase is basically determined only by the single state variable [48]. The two types of phase resetting and a change of type depending on the pulse strength, however, can be theoretically explained by the concept that a clock is an endogenous oscillator that has two or more mutually interacting state variables. Especially, phase responses of circadian clocks have often been explained in terms of the limit cycle model [31,48,49]. In this model, two or more state variables of the circadian clock oscillate in phase space around a trajectory of the limit cycle. The central portion of the limit cycle is called the point of singularity. A circadian phase is uniquely determined by the oscillating state variables, and changes of the state variables in phase space are provoked by the resetting stimuli. The magnitude of the changes depends on the strength of the stimuli. In the middle of the subjective night, Type 0 (or strong) resetting stimuli move the state variables beyond the point of singularity and then strikingly induce a phase shift, whereas Type 1 (or weak) stimuli do not move the state variables beyond the point of singularity and consequently induce little or no phase shift.

**Figure 2:**
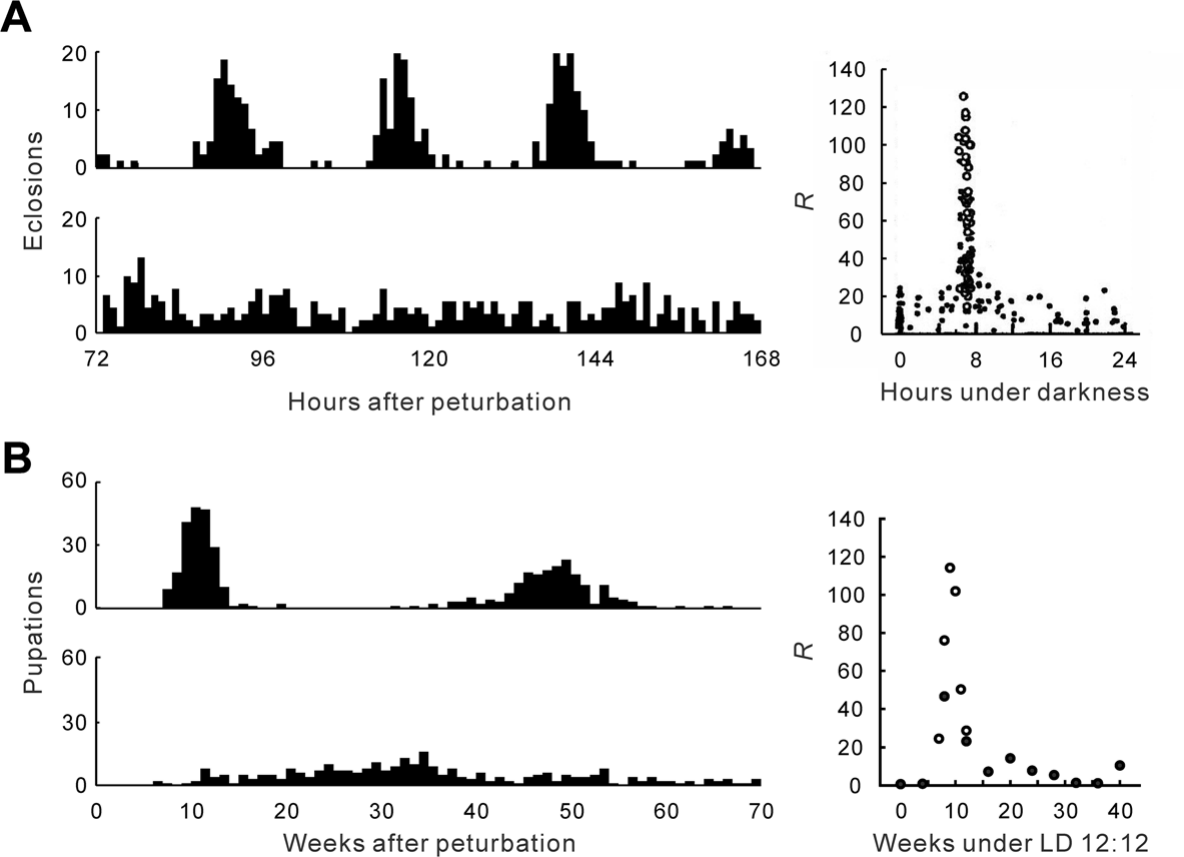
**Induction of arrhythmicity by a specific stimulus in circadian and circannual clocks. (A)** Circadian eclosion rhythm in *Drosophila pseudoobscura*. Arrhythmicity was induced with a blue-light pulse at an intensity of 0.1 W/m^2^ near conditions of a pulse length of 50 s and of a time of pulse onset of 6.8 h after transfer to constant darkness (lower left), whereas without pulses, circadian rhythmicity was shown in constant darkness (upper left) [42]. The right panel shows the arrhythmicity induced by a blue-light pulse (0–120 s). *R* values were calculated from hourly emergence counts in three or four circadian cycles and are plotted as a function of the time of pulse onset. The larger circles represent the experiments using stimuli particularly close to conditions of a pulse length of 50 s and of a time of pulse onset of 6.8 h after transfer to constant darkness (reproduced from [42] with kind permission from Elsevier). **(B)** Circannual pupation rhythm in *Anthrenus verbasci*. Arrhythmicity was induced with a 4-week long-day pulse of LD 16:8 applied nine weeks after exposing larvae to LD 12:12 (lower left), whereas without pulses, circannual rhythmicity was shown with a period of approximately 40 weeks under constant short-day conditions of LD 12:12 (upper left) (reproduced from [47] with kind permission from Springer Science+Business Media). The right panel shows the arrhythmicity induced by a 4-week long-day pulse. *R* values were calculated from weekly pupal counts in one or two circannual cycles (closed circles [46]), or three circannual cycles (open circles [47]) and are plotted as a function of the time of pulse onset. Statistically complete arrhythmicity is shown by *R* values approaching approximately 150 [42]. In practice, *R* values of 30 or less are considered highly rhythmic and those greater than 90 arrhythmic [2,42,47].

In the eclosion rhythm of *D. pseudoobscura*, Winfree [42] found that arrhythmicity was induced when a blue-light pulse of intermediate strength (50 s) was applied at the phase of transition between delays and advances in the PRC, i.e., near the middle of subjective night (Figure 2A). Similar results have been obtained for circadian rhythms of other insects [44,50-52]. These phenomena can be explained by the notion that state variables of the clock are changed to the region close to the point of singularity and the clock is driven to the phaseless state or populations of oscillators are desynchronized. These singular behaviors, in addition to Type 0 phase resetting, cannot be explained if a clock is a one-dimensional oscillator [31,42,48,49].

### Circadian clock for celestial navigation

Celestial navigation generally needs to be time-compensated by a circadian clock and, therefore we assume that the PRC of this clock is similar to PRCs of circadian clocks for daily rhythmic events. More than half a century ago, similarity of phase changes between the circadian clocks involved in the locomotor activity rhythm and solar compass was noted in the European starling, *Sturnus vulgaris* [53]. In *D. plexippus*, a phase delay or advance of the circadian clock by artificial light–dark cycles produces a change in orientation in solar compass navigation, as would be caused by a phase difference in the circadian clock [5,54]. In *A. mellifera*, general anesthesia for 6 h in daytime delayed the phase of the circadian rhythms in foraging time, locomotor activity in hives, and clock gene expression, and changed the orientation with an anticlockwise shift in the Southern Hemisphere and with a clockwise shift in the Northern Hemisphere in solar compass navigation [55]. Moreover, nighttime anesthesia produced little or no phase shift in locomotor activity, and therefore, a PRC for anesthesia might be obtained in these rhythms. However, only daytime anesthesia was used for solar compass orientation [55], and a complete PRC for the clock for celestial navigation in insects remains to be identified.

### Circadian clock for photoperiodism

It is now accepted that a circadian clock is involved in photoperiodism [11-13]. In fact, a major theoretical model for photoperiodic time-measurement, the external coincidence model, was developed to explain the photoperiodic induction of diapause in the pink bollworm, *Pectinophora gossypiella*, with reference to the PRC of the circadian eclosion rhythm in *D. pseudoobscura* [41]. In this model, light plays two roles: (1) the entrainment and phase resetting of the circadian clock involved in photoperiodism, and (2) the coincidence of light with the photo-inducible phase (φ_i_) which is assumed to occur in the subjective night [41].

Saunders [43] compared PRCs between the circadian eclosion rhythm and photoperiodic induction of pupal diapause in the same species, *S. argyrostoma*, in which the photoperiodism is explained by the external coincidence model. To construct a PRC for the circadian clock for photoperiodism, Saunders [43] designed 3-point skeleton photoperiods of 72 h with a 12-h main light component, a 15-min resetting pulse applied at different phases in the first subjective night, and a 1-h scanning pulse for φ_i_ in the second subjective night. Using this protocol, phase shifts of φ_i_ caused by 15-min light pulses were detected, and a PRC for the circadian clock for photoperiodism was obtained (Figure 1C). Saunders [43] emphasized the similarities in PRCs between the circadian clocks for eclosion rhythm and photoperiodism.

### Circatidal clock

To investigate the effects of inundation on the circatidal rhythm of *A. asahinai*, Satoh et al. [45] provided an inundation pulse (0.5 h) four times at intervals of 12.4 h in constant darkness and temperature and observed the entrainment of this circatidal rhythm to periodic inundations. A circatidal PRC was constructed by plotting phase shifts as a function of the phase of the first inundation onset. Periodic inundations during the first half of the subjective low tide of the circatidal cycle caused phase delays, whereas those during the second half of the subjective low tide caused phase advances, and those during the subjective high tide produced only small phase shifts (Figure 1D) [45]. These responses are similar to those caused by light pulses in the circadian rhythm, but the responsiveness changes with semi-diurnal tidal periodicity. The cross over point between delays and advances occurred near the middle of the subjective low tide in the PRC of *A. asahinai*, which is a common characteristic of Type 1 PRCs of circadian rhythms. It has long been controversial whether the clock mechanism producing circatidal rhythms is a circatidal clock or a tidally adapted circadian clock(s) [20,56-58]. The similarity between the circadian and circatidal PRCs indicates that an underlying oscillator of the circatidal rhythm of *A. asahinai* is similar to a circadian clock in phase responses, but its period is approximately 12.4 h. Thus the circatidal rhythm of this species is controlled by a circatidal clock [45].

### Circalunar and circasemilunar clock

Artificial moonlight is effective as a Zeitgeber for many circalunar or circasemilunar rhythms [25,56]. In the circasemilunar rhythm of *C. marinus*, for example, distinct entrainment can be achieved by exposure to artificial moonlight not only every 30 days but also every 24 or 36 days, suggesting that phase shifts are elicited by exposure to artificial moonlight in a phase-dependent manner [24].

Although the PRC of a circalunar clock for artificial moonlight was shown in the syllid polychaete *Typosyllis prolifera* [59], no PRCs for circalunar or circasemilunar clocks have been obtained in insects. *C. marinus* shows a circalunar or circasemilunar rhythm depending on the strain [16,60]. It is unknown whether circalunar and circasemilunar rhythms of *C. marinus* are derived from the same oscillator with different output forms or from different oscillators with different periods [61]. The construction of PRCs for artificial moonlight may be one approach to addressing this question.

### Circannual clock

A change in photoperiod is a predominant Zeitgeber in a circannual pupation rhythm of *A. verbasci* [15]. To construct a PRC for this rhythm, Miyazaki et al. [46] kept *A. verbasci* larvae under short-day conditions of 12-h light and 12-h darkness (LD 12:12) and exposed to LD 16:8 for four weeks (4-week long-day pulse) during various phases of the rhythm. A 4-week long-day pulse markedly shifted the phase of the circannual rhythm. Whether this pulse advanced or delayed the circannual phase depended on the phase in which the pulse was given. A long-day pulse applied in early subjective winter of the circannual cycle delayed the phase, whereas a pulse in late subjective winter advanced the phase. A long-day pulse applied in subjective summer had relatively little effect on the phase. A PRC to 4-week long-day pulses was constructed based on these observations (Figure 1E). This circannual PRC closely resembles the Type 0 PRC of circadian rhythms (Figure 1A). Thereafter, Miyazaki et al. [47] constructed a circannual PRC to 2-week long-day pulses (Figure 1F). The phase shifts caused by 2-week pulses were smaller than those caused by 4-week pulses. The PRC to 2-week long-day pulses had a continuous transition between delays and advances in the middle of the subjective winter. Therefore, this curve is categorized as Type 1.

Thus, the circannual pupation rhythm of *A. verbasci* shows similarity in the phase response to Zeitgeber stimuli with the circadian eclosion rhythm of *D. pseudoobscura*. The general characteristics of biological clocks discovered in the circadian rhythm of *D. pseudoobscura* can be useful for clarifying fundamental properties of the circannual rhythm of *A. verbasci* [15,46]. In the circadian rhythm of *D. pseudoobscura*, arrhythmicity could be induced when a blue-light pulse of 50 s was applied at the phase of transition between delays and advances in the PRC [42] (also see above). Similarly, Miyazaki et al. [47] showed that a 4-week long-day pulse administered at the phase of transition between delays and advances in the circannual PRC, i.e., near the middle of subjective winter, could evoke arrhythmicity (Figure 2B). These results suggest that the theoretical basis of oscillation in the circannual rhythm in *A. verbasci* has many remarkable parallels to that of the circadian rhythm in *D. pseudoobscura*.

Three hypotheses have been proposed for the physiological mechanism that generates a circannual rhythm [62,63]. The first hypothesis is that a circannual rhythm is generated by a self-sustaining biological clock with a period of approximately one year, i.e., a circannual clock, analogous to the circadian clock. The second hypothesis is that a circannual rhythm is derived from a circadian rhythm through a process of frequency demultiplication [18], and the third hypothesis is that a circannual rhythm merely results from a sequence of some linked physiological stages of which the last stage is linked back to the first [62]. Although the latter two hypotheses may explain the mechanism for generation of the circannual rhythm as an extension of well-known physiological mechanisms, no direct experimental results have supported them [23,64]. In addition, because the physiological mechanisms predicted by these two hypotheses are one-dimensional oscillators whose phase is determined by the single state variable, they cannot explain Type 0 phase resetting and arrhythmicity in the circannual rhythm of *A. verbasci* [23,48]. According to the theoretical explanations, for Type 0 and Type 1 phase resetting and the loss of rhythmicity induced by a Zeitgeber pulse [48] (also see above), the mechanism behind the circannual rhythm of *A. verbasci* is thought to be an endogenous oscillator with two or more mutually interacting state variables with circannual variation. This indicates that the circannual rhythm of *A. verbasci* is generated by a circannual clock that has a period of approximately one year and that has characteristics in common with those of the circadian clock [23,46,47].

### Anatomical locations

#### Circadian clock for daily rhythmic events

**Figure 3:**
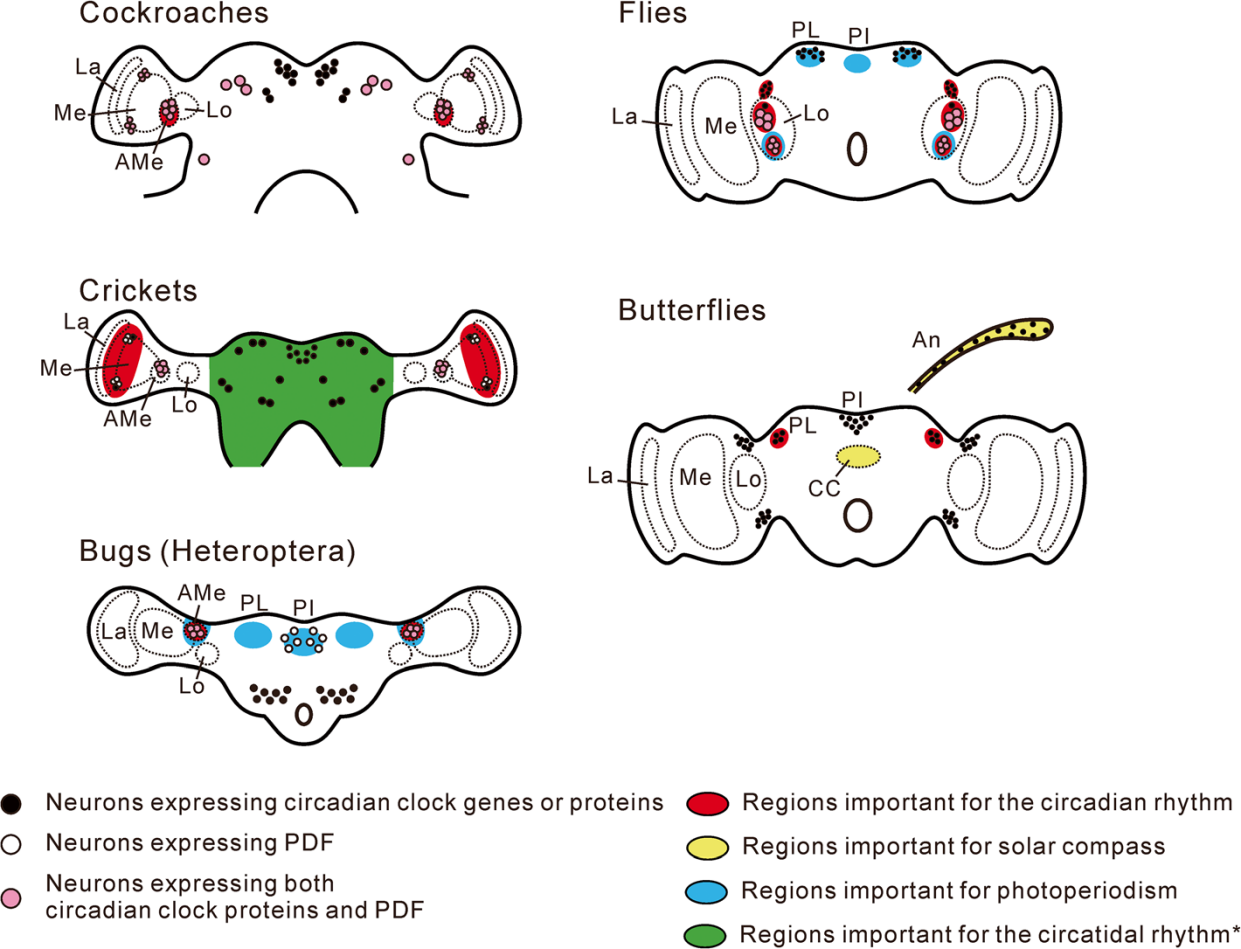
**Neurons or regions important for the circadian rhythm, solar compass, photoperiodism, and circatidal rhythm.** In cockroaches, the accessory medulla containing PDF-expressing neurons is the location of the circadian clock regulating activity rhythms [71,73]. In crickets, the circadian clock is located in the lamina and medulla regions [74]. *It is likely that the circatidal clock is not located in the optic lobe, but is probably located in the central brain [28], although the possibility that the clock is located in an extra-brain region cannot be excluded. In *Rhodnius prolixus* (Heteroptera), lateral neurons, which co-express clock proteins and PDF, are considered to be the circadian clock that regulates the activity and hormone-release rhythms [75,76]. The region containing these neurons is involved in the photoperiodic diapause in another heteropteran, *Riptortus pedestris* [77]. The PI and PL regions are also important for photoperiodic diapause in heteropterans [78,79]. In flies, among neurons expressing clock proteins in the brain, several groups of lateral neurons control the circadian activity rhythm. One group of neurons that co-express clock proteins and PDF is important for photoperiodism [80]. Neurosecretory cells in the PI and PL regulate photoperiodic diapause [81]. In butterflies, the PL is the main location of the circadian clock in the brain [82,83]. Neurons necessary for the solar compass are located in the central complex, and the circadian clock in the antennae is necessary for time compensation of the solar compass system [84,85]. Note that the neuronal location may differ among species. La, lamina; Me, medulla; AMe, accessory medulla; Lo, lobula; PI, pars intercerebralis; PL, pars lateralis; CC, central complex; An, antenna.

Circadian clocks have been localized either in the central nervous system or in peripheral tissues. The circadian clocks in the central nervous system, i.e., the central clocks, are localized in the optic lobe or mid-brain (Figure 3) [65]. The optic lobe flanks the mid-brain, a medial part of the brain, and contains the neuropils involved in the preprocessing of visual information from the compound eye. In cockroaches and crickets, the circadian clock in the optic lobes controls their activity rhythm [33-35,66-70]. The optic lobe consists of lamina, medulla, and lobula complex. In the cockroach *R. maderae*, it was shown by transplantation experiments that the accessory medulla, a small neuropil situated at the ventromedial edge of the medulla, is the locus of the circadian clock [71]. In the cricket *G. bimaculatus*, the medulla and the lobula are connected by the optic stalk. Removal of the lamina and medulla regions results in arrhythmic locomotion activity [72]. Furthermore, efferent electrical activities of the neurally isolated lamina and medulla recorded from the optic stalk show clear circadian changes [68]. In *G. bimaculatus*, therefore, the circadian clock should be in the lamina or medulla region, although more precise localization has not been determined.

In Lepidoptera and Diptera, the circadian clock is localized in the mid-brain. In saturniid moths *Antheraea pernyi*, *Hyalophora cecropia* and *Samia cynthia*, the circadian clocks for the eclosion and flight rhythms are localized in the mid-brain [86-88]. In *A. pernyi*, two pairs of neurosecretory cells in the dorsal part of the mid-brain have been shown to express the clock protein PERIOD (PER), and these cells are regarded as the site of the circadian clock [89]. In studies on the circadian clock of *D. melanogaster*, genetic analyses advanced first (see below), and based on those analyses, the brain neurons expressing the clock genes and proteins became candidates for the circadian clock. Approximately 150 neurons in the mid-brain express clock genes or proteins, and are traditionally divided into six groups based on their location [90]. Each group appears to have a different role in the neuronal network to regulate behavioral rhythms, but some groups of lateral neurons have been shown to be the main locus of the circadian clock controlling the locomotor activity rhythm [91-93]. Among these circadian clock neurons, all large lateral ventral neurons and four of five small lateral ventral neurons express a neuropeptide, the pigment-dispersing factor (PDF), which plays dual roles in synchronizing molecular oscillations of circadian clock neurons [94] and in the output pathway from the circadian clock [95].

In addition to the central clocks, circadian clocks reside in various peripheral tissues, e.g., prothoracic glands, testes, Malpighian tubules, and epidermal cells, and control physiological functions of these tissues [40,96-100].

Cells expressing circadian clock components have been identified in various insects in addition to *D. melanogaster*, e.g., *D. plexippus*, the blow fly *Protophormia terraenovae*, and the bloodsucking bug *Rhodnius prolixus* [75,76,80,82,83]. However, it is difficult to determine whether these cells are the location of circadian clocks, because in these insects genetic tools are usually unavailable, and it is almost impossible to examine the function of specific cells. Indeed, some clock genes have been shown to be involved in non-rhythmic phenomena that are not related to the clock system [101-104]. Therefore, the newly identified cells expressing circadian clock components have in many cases been subjected to several further tests, such as anatomical comparison with already identified circadian clock cells in other species and examination of temporal expression patterns of clock genes within the cells and co-expression of other clock genes. Nevertheless, we must be careful about judging whether these cells function as circadian clocks.

### Circadian clock for celestial navigation

Similarities in the phase response between the circadian clocks for behavioral rhythms and time-compensated celestial navigation suggest that the same clock in the brain is used for these two behaviors [55] (see above). Sauman et al. [82] suggested that in *D. plexippus*, neurons in the pars lateralis (PL) of the protocerebrum expressing a circadian photoreceptor, CRYPTOCHROME (*Drosophila*-type CRY, see below), as well as core clock proteins PER and TIMELESS (TIM), are the locus of the circadian clock for time-compensated celestial navigation, because they have a connection to the photoreceptor cells in the dorsal rim area, and these are specialized for polarized light, which is one of the celestial cues for solar compass orientation. However, a peripheral clock also plays an important role in solar compass orientation of this species. The light-entrained circadian clock in the antennae is necessary, and integration between the bilateral antennal clocks is important, for correct orientation [84,85]. Merlin et al. [84] proposed that interaction between the antennal and brain circadian clocks is important for solar compass orientation, although the predominant role of the antennal clock is independent of the brain clock. Solar compass orientation of the desert locust, *Schistocerca gregaria*, requires time compensation for changes in solar elevation in addition to the azimuthal compensation [105]. Heinze and Reppert [106] suggested that in *D. plexippus*, the solar compass mechanism involves two distinct circadian clocks, i.e., the azimuthal and elevation compensations are regulated by circadian clocks in the PL and the antennae, respectively (Figure 3).

### Photoperiodic clock

The simplest model for photoperiodism contains three functional components: (1) a photoreceptor to distinguish light from dark, (2) a photoperiodic clock to measure the length of the day or night, and (3) an endocrine effector to regulate a seasonal response, such as diapause [2]. Experiments with transplantation or *in vitro* culture have shown that all of these three components are in the brain or brain-subesophageal ganglion-complex in larvae or pupae of lepidopterans [107-109]. Moreover, a diapause pupa of *A. pernyi* responded to photoperiod after its brain was excised and the dorsal half of a mid-brain was implanted, suggesting that the three components, including the photoperiodic clock, reside in this region [110].

When surgical removal or cauterization of a certain region of the brain abolishes photoperiodism, it is considered that at least one of the three components resides in the removed or cauterized region. Such results have been obtained in some insects [78,79,81,111]. In the vetch aphid, *Megoura viciae*, destruction of the pars intercerebralis (PI), an anterior dorsal region of the protocerebrum including medial neurosecretory cells, abolished the photoperiodism [112]. Steel and Lees [112] assumed that these neurosecretory cells and a brain region slightly lateral to these cells are the endocrine effector and the photoperiodic clock, respectively. By surgical removal or cauterization alone, however, it is impossible to identify which of the three components for photoperiodism resides in the operated region. *P. terraenovae* enters adult diapause with suppression of ovarian development under short-day conditions. In this species, not only surgical removal of the PI but also bilateral removal of the PL, a region of the protocerebrum slightly lateral to the PI, disrupts the photoperiodism. The former suppresses ovarian development, whereas the latter prevents diapause irrespective of photoperiod [81,111]. Both the PI and PL are in the protocerebrum and neurally connected to the corpus allatum (CA), the major endocrine organ for the control of adult diapause in this species [113]. It is therefore thought that these brain regions contain the endocrine effector for the photoperiodism or its regulatory neurons (Figure 3). In the bean bug *Riptortus pedestris*, surgical removal of the PL disrupts photoperiodism in the induction of adult diapause as in *P. terraenovae*, although the PI is not necessary for ovarian development, in contrast to its necessity in *P. terraenovae* [79]. Based on these results, Shimokawa et al. [79] suggested that the PL inhibits the CA activity in photoperiodic diapause. In the brown-winged green bug, *Plautia stali*, whose adult diapause involves low juvenile hormone (JH) biosynthetic activities, results by *in vitro* experiments of the CA and brain neuroanatomy showed that neurons with somata in the PI or PL are candidates for inhibition of the CA activity in diapause [114].

Formal experiments such as those with Nanda-Hamner and Bünsow protocols have shown that the circadian system is involved in physiological mechanisms underlying photoperiodism [2,11,13,115,116], implying that the photoperiodic clock contains a circadian clock, or is closely connected to it. Thus, to localize the photoperiodic clock one plausible approach is to examine the role of the circadian clock neurons for daily rhythmic events in photoperiodism. In the tobacco hornworm, *Manduca sexta*, surgical ablation of PL neurons expressing *per* results in the loss of photoperiodism for the induction of pupal diapause, although it is unknown whether these neurons play roles in circadian rhythms [117]. Given that in *D. plexippus* the circadian clock for celestial navigation is also considered to be located in PL neurons [82], it is likely that PL neurons expressing clock genes or proteins are important for the control of various circadian clock-related phenomena in Lepidoptera. In *P. terraenovae*, Shiga and Numata [80] focused on the brain neurons expressing both PER and PDF that were shown to be the site of the circadian clock in *D. melanogaster* (see above). After surgical removal of small lateral ventral neurons, most flies became behaviorally arrhythmic, and also lost photoperiodism for adult diapause (Figure 3) [80]. In *R. pedestris*, surgical removal of the region containing PDF-immunoreactive somata at the anterior proximal medulla of the optic lobe disrupted the photoperiodic regulation of diapause [77]. Ikeno et al. [77] suggested that this region is important for the photoperiodism. However, the role of these neurons in circadian rhythms of *R. pedestris* remains unclear, although these neurons co-express clock proteins and have been suggested to be circadian clock neurons that regulate rhythmic hormonal secretion in another heteropteran insect, *R. prolixus* (Figure 3) [75,76].

Hypotheses that a peripheral tissue contributes to photoperiodism involving the tissue’s interaction with a brain center were proposed many years ago [118,119], although no reliable experimental results have supported them. Recently Bajgar et al. [120] showed that four clock genes in the gut play important roles in photoperiodic switching of reproductive and diapause status of the gut in the linden bug, *Pyrrhocoris apterus*. In the gut of this species, expression of these clock genes does not show circadian oscillation, but rather is controlled by endocrine signals from the CA [121,122]; these genes thus do not seem to be components of a circadian clock in the gut.

### Circatidal clock

Physiological mechanisms generating circatidal rhythms have been examined mostly in crustaceans, and the results show that the circatidal clock or neurons under its control reside in the eyestalk [123,124]. In insects, Takekata et al. [28] have recently examined the location of the circatidal clock in *A. asahinai*. This cricket living on the floor of mangrove forests shows a circatidal activity rhythm generating active and inactive phases and a circadian rhythm that modifies the circatidal rhythm by inhibiting activity during the subjective day. After the removal of the optic lobes, the circadian modulation was disrupted but the circatidal rhythm was maintained with no remarkable changes in its free-running period. Therefore, the circatidal clock is located in a region(s) different from the optic lobe, whereas the circadian clock is located in the optic lobe, as in other crickets (Figure 3) [70].

### Circalunar and circasemilunar clocks

Fleissner et al. [125] suggested that the larval ocelli of *C. marinus* function as moonlight receptors for entrainment of the circalunar clock, and are also controlled by the circalunar clock itself, based on the finding that the shielding pigment granules in larval ocelli changed reversibly between black-brown and transparent during lunar phases. Therefore, the larval ocelli are primary candidates for use in the identification of the circalunar clock by tracing input and output pathways of the circalunar clock. Although Fleissner et al. [125] considered that a lunar-rhythmic change in the larval ocelli provided a possible handle for approaching the neuronal basis of the circalunar (or circasemilunar) clock, further reports on the location of the clock in *C. marinus* have not appeared.

### Circannual clock

The exact anatomical location of the mechanism that generates circannual rhythmicity is not clear in any organism, although it has been suggested that in sheep, *Ovis aries*, the adenohypophysis is the site of the circannual pacemaker for prolactin secretion [126]. Miyazaki et al. [127] showed with the Nanda-Hamner protocol that the circadian system is involved in photoperiodic entrainment of the circannual rhythm in *A. verbasci*. Therefore, the circadian clock for photoperiodism must be closely related to the circannual clock in this species. Identification of the anatomical location of a circannual clock of *A. verbasci* will be aided by reference to the centers for photoperiodism and circadian rhythm [23].

### Clock genes

#### Circadian clock for daily rhythmic events

**Figure 4:**
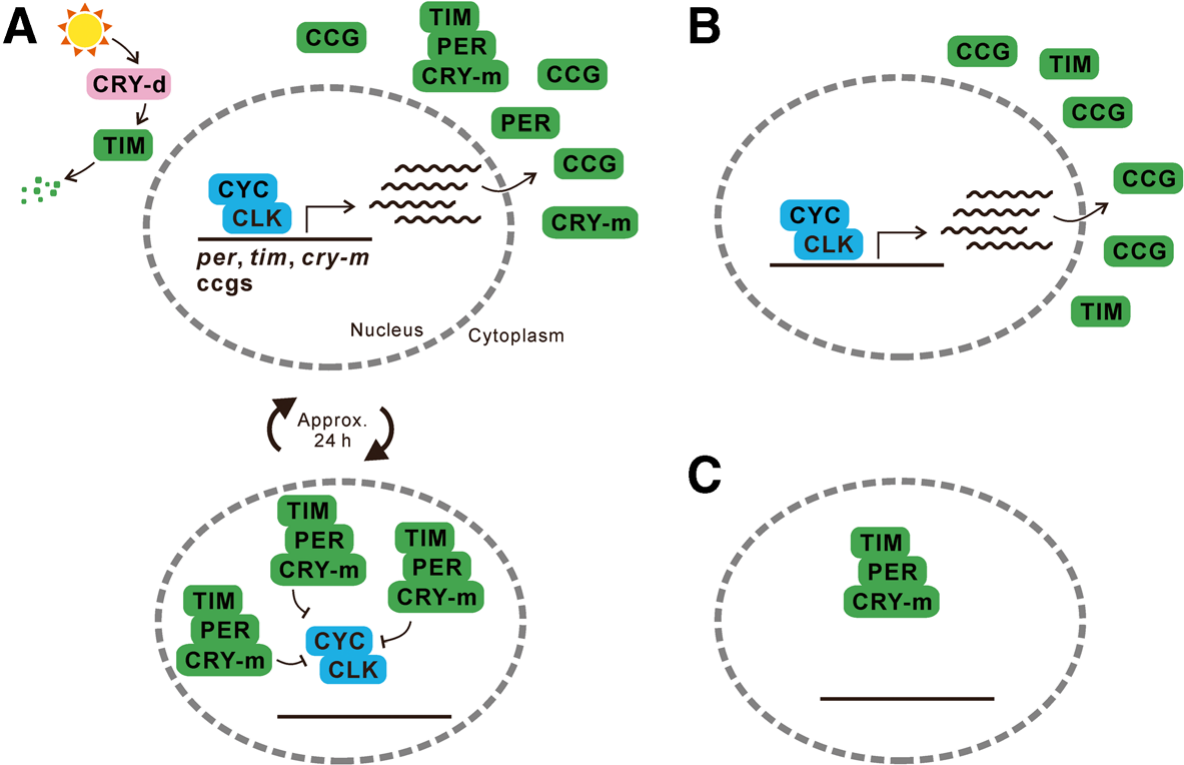
**Molecular mechanisms of the circadian clock and effects of clock gene RNAi on the circadian clock in** ***Riptortus pedestris*** **. (A)** A general model of the insect circadian clock mechanism. Positive elements of CYC and CLK form a heterodimer and activate transcription of clock genes *per*, *tim*, and *cry-m* and many other genes called clock-controlled genes (*ccg*). Their protein products are synthesized in the cytoplasm and PER, TIM and CRY-m form a complex. In *Drosophila melanogaster*, only PER and TIM form a heterodimer. The complex enters the nucleus and acts as a negative element that represses CYC/CLK transcriptional activity. Reduction of *ccg* transcript levels and consequent reduction of CCG protein levels lead to a decrease of repressive regulation of CYC/CLK by PER/TIM/CRY-m, and therefore CYC/CLK-mediated transcription increases again. These phases in which *ccg* transcription is activated or repressed are repeated in approximately 24 h. CRY-d causes degradation of TIM in a light-dependent manner. **(B)** When negative elements PER and CRY-m are eliminated by RNAi in *R. pedestris*, the circadian clock remains at the phase in which *ccg* transcription is activated [129]. **(C)** When positive elements CYC and CLK are eliminated, the circadian clock remains at the phase in which *ccg* transcription does not occur [130]. Note that the absence of transcriptional activity would also decrease the protein levels of negative elements.

The molecular basis of the circadian clock has been clarified predominantly in *D. melanogaster*. Although the clock involves at least three interlocked transcriptional and translational feedback loops, here we describe only the major loop consisting of four clock genes, i.e., *per*, *tim*, *Clock* (*Clk*), and *cycle* (*cyc*) (Figure 4A) [37-39]. In this loop, the product proteins of *Clk* and *cyc*, CLK and CYC, respectively, form a heterodimer to activate transcription of *per*, *tim*, and clock-controlled genes, and therefore they are referred to as positive elements. On the other hand, the products of *per* and *tim*, PER and TIM, respectively, also form a heterodimer and function as negative elements by suppressing the activity of CLK/CYC for transcription of *per*, *tim*, and clock-controlled genes. Consequently, the phases with high and low expression of *per*, *tim*, and clock-controlled genes and their protein products change periodically with a period of approximately 24 h, producing circadian rhythms in daily events. In addition to the roles of the retinal receptors (compound eyes and ocelli), and Hofbauer-Buchner eyelets [128], a blue- and UV-light receptor protein, *Drosophila*-type CRY (CRY-d), plays a role in photic entrainment of the circadian clock by causing a degradation of TIM in a light-dependent manner.

The basic feedback regulation of the circadian clock provided by positive and negative elements is highly conserved among insects; nevertheless, there is also significant diversity of the molecular mechanism among insect circadian clocks, and the structure revealed in *D. melanogaster* cannot simply be applied to those in other insects [37]. Although *D. melanogaster* has only one *cry* gene (*cry-d*, called *cry1* also) in its genome, many other insects, such as *D. plexippus,* have an additional type of *cry*, which is called mammalian-type *cry* (*cry-m*, called *cry2* also), as it is a homologue of *cry* in mammals [83,131]. By contrast to CRY-d, CRY-m does not function as a photoreceptor but contributes to the circadian feedback loop as a negative element by forming a complex with PER and TIM to suppress the CLK/CYC activity [83,131]. It should be noted that the *tim* gene is absent from the genome of *A. mellifera*, which also lacks the *cry-d* gene [132].

### Circadian clock for celestial navigation

In *A. mellifera*, general anesthesia for 6 h in daytime has a similar phase-shifting effect on *per* and *cry-m* expression in the brain, the phase of the circadian behavioral rhythms, and the orientation in solar compass navigation [55]. From these results, Cheeseman et al. [55] concluded that the circadian clocks for daily events and celestial navigation have a common molecular basis. In *D. plexippus*, the expression of clock genes *per*, *tim*, and *cry-m* shows circadian changes in the antennae that are necessary for solar compass orientation [84]. However, there is no direct evidence that these clock genes are involved in time compensation for celestial navigation in these species.

### Photoperiodic clock

A straightforward step toward clarifying the molecular mechanism of photoperiodism seemed to be examining the role of clock genes in *D. melanogaster*, because the circadian system had been shown to be involved in photoperiodism, and clock genes had been extensively identified in *D. melanogaster* (see above). However, *D. melanogaster* had long been accepted as an insect without photoperiodism. Saunders and his co-workers first showed that this species actually has a photoperiodically controlled adult diapause and then examined the photoperiodism in an arrhythmic mutant, *per*^*0*^ [133,134]. This mutant also showed photoperiodism even though the critical daylength was shorter than that in wild-type flies. Therefore, these authors suggested that *per* is not causally involved in the photoperiodism of *D. melanogaster*. However, the diapause in this species is weak and variable, and the photoperiodic effect is shown only in a narrow temperature range. These results have yet to be reproduced.

In *D. melanogaster*, flies possessing a long TIM isoform that provides a circadian clock with lower photosensitivity show higher diapause incidence under various photoperiods, suggesting that *tim* is associated with photoperiodic induction of diapause [135,136]. In *Drosophila triauraria*, genetic crosses between a strain showing a clear photoperiodism for adult diapause and a non-diapause strain revealed that allelic differences in *tim* and *cry-d* between the strains were additively associated with the differences in the incidence of diapause [137]. In another drosophilid fly, *Chymomyza costata*, *tim* has been shown to be crucial for photoperiodic control of larval diapause. A strain with mutated *tim*, the non-photoperiodic-diapause (*npd*) mutant, does not show either circadian eclosion rhythm or photoperiodism to control larval diapause [138-140]. Moreover, the *npd*-mutant has a 1855-bp-long deletion in the 5′-UTR region of *tim*, and larvae of this mutant show lower levels of *tim* transcription and TIM accumulation than wild-type larvae [141]. All these results indicate the involvement of *tim* in photoperiodic induction of diapause, but in *D. melanogaster* the diapause-enhancing effect of *tim* occurs not through the photoperiodic clock but through an interaction with *cry-d* as a circadian photoreceptor [135].

Recently, Pegoraro et al. [142] focused on chill-coma recovery times regulated by photoperiodism in *D. melanogaster*. Whereas wild-type flies raised under short days exhibited shorter recovery times than flies raised under long days, null mutants of clock genes, *per*^*01*^, *tim*^*01*^, and *Clk*^*JRK*^, did not show the photoperiodic response, suggesting that the clock genes play an important role in photoperiodism [142]. Moreover, clock mutants with long circadian periods showed shorter recovery times than mutants with short circadian periods, irrespective of photoperiod. Pegoraro et al. [142] explained these results with the external coincidence model, and consider them as the first evidence in *D. melanogaster* to support the Bünning’s hypothesis.

RNA interference (RNAi) enabled the use of loss-of-function analyses in many non-model insects to elucidate the roles of specific genes [143,144]. In the cricket *Modicogryllus siamensis*, which shows a long-day photoperiodic response for nymphal development, maternal RNAi of *per* resulted in a moderate development rate both under long-day and short-day conditions, like the rate in control insects in constant darkness [145]. Thus, *per* plays an essential role in photoperiodic control of nymphal development in this species. In *C. costata*, in which the critical role of *tim* in photoperiodism was first shown by analyses of a mutant strain (see above), RNAi of *tim* prevented diapause under diapause-inducing short-day conditions in a small proportion of larvae, but a few control larvae with injection of buffer only also lacked diapause, and thus no conclusion could be drawn about the effect of *tim* RNAi [140].

Although it is probable that some clock genes play roles in photoperiodism, it is unclear how and where these genes are involved in it. The key question here is whether these clock genes act in photoperiodism in a form of the circadian clock or through their non-clock pleiotropic functions [146]. Non-clock functions of clock genes have been widely demonstrated in various insects [101-104]. Indeed, the photoperiodic regulation of the gut status by clock genes in *P. apterus* seems to be independent of their functions in the circadian clock [120,122]. A series of studies by Ikeno et al. [129,130,147,148] applying RNAi of clock genes in *R. pedestris* approached this issue and revealed that the circadian clock composed of clock genes plays an important role in the photoperiodic regulation of reproductive diapause. When the expression of the *cyc* gene was suppressed by RNAi, insects failed to develop their reproductive organs and entered diapause even under diapause-preventing long-day conditions [130,147]. Later, it was shown that RNAi of the other positive element, *Clk*, also induced the same phenotype, i.e., diapause under long-day conditions [148]. On the other hand, gene suppression of negative elements, *per* and *cry-m*, led to development of reproductive organs even in diapause-inducing short-day conditions [129,130,147]. These findings suggest not only that clock genes are involved in the photoperiodic response but also that the positive and negative elements have opposite roles in the response mechanism.

**Figure 5:**
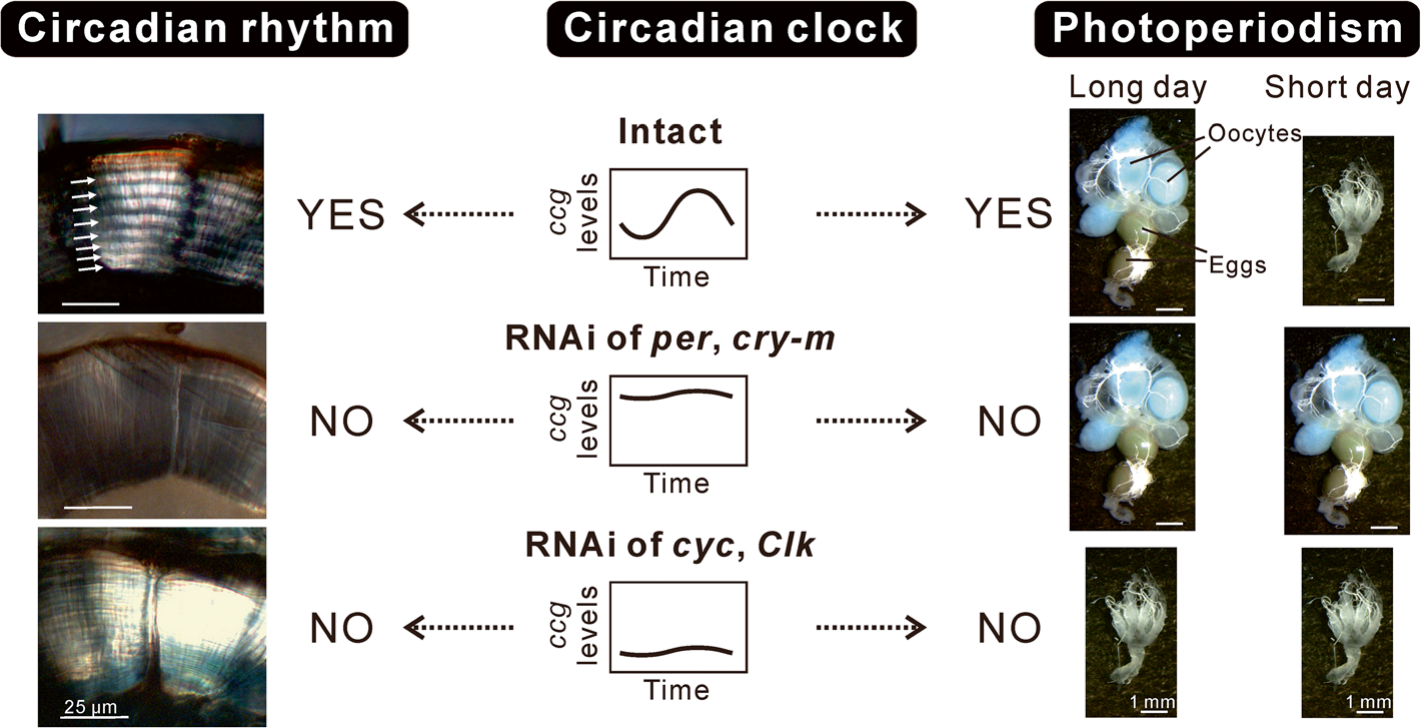
**A hypothetical explanation of clock gene RNAi in** ***Riptortus pedestris*** **.** The same molecular machinery is required for the circadian rhythm of cuticle deposition and the photoperiodic response [129,130,147-149]. In the intact group, the circadian clock generates rhythmic transcription of *ccg*, regulating the circadian rhythm with alternating deposition of polarized cuticle layers and nonpolarized cuticle layers, which are observed as bright (indicated by small arrows) and dark layers, respectively, under a light microscope with crossed polarizers. The intact group shows a clear photoperiodic response: Female adults develop their ovaries under long-day conditions but enter diapause under short-day conditions. In the *per* or *cry-m* RNAi group, constant high levels of *ccg* transcripts abolish the circadian rhythm of cuticle deposition, and only nonpolarized layers are deposited. Photoperiodism is also disrupted and ovarian development is induced irrespective of the photoperiod. In the *cyc* or *Clk* RNAi group, constant low levels of *ccg* transcripts abolish the circadian rhythm and only polarized layers are deposited. In this case as well, photoperiodism is disrupted and diapause is induced irrespective of the photoperiod.

Simultaneously, the opposite effects caused by RNAi of positive and negative elements on the circadian clock function were confirmed by observation of the circadian rhythm in cuticle deposition. After adult emergence, the endocuticle of *R. pedestris* thickens by an alternating deposition of polarized and nonpolarized cuticle layers [130], and this rhythm was shown to be regulated by the peripheral clock in the epidermis in *D. melanogaster* [100]. RNAi of *cyc* and *Clk* prevented the rhythmic switching of the two layers and resulted in the deposition of only polarized layers [130,148]. By contrast, RNAi of *per* and *cry-m* also disrupted the cuticle deposition rhythm but resulted in the deposition of only nonpolarized layers [130,149]. Considering the molecular mechanism of the circadian clock, these opposite effects of positive and negative elements are reasonable: suppression of *per* and *cry-m* would induce constant transcriptional activation by CYC/CLK and “stop” the clock at the phase of high expression of clock-controlled genes, resulting in deposition of only nonpolarized layers (Figure 4B). By contrast, suppression of *cyc* and *Clk* would abolish the transcription of clock-controlled genes and “stop” the clock at the phase of low or no expression of clock-controlled genes, resulting in deposition of only polarized layers (Figure 4C). Indeed, it was demonstrated that *per* mRNA expression was decreased by *cyc* RNAi [130], whereas it was increased by *cry-m* RNAi, in *R. pedestris* [129]. Given these effects of RNAi of clock genes on the clock mechanism, it is reasonable to consider that being in different phases when the clock stopped also induced different phenotypes for photoperiodic diapause, suggesting that the cyclic phase change of the clock is required for photoperiodism (Figure 5).

Where then, does the circadian clock function in the photoperiodic system? Opposing effects of *per* and *cyc* RNAi on the photoperiodism in *R. pedestris* were observed not only in ovarian development of females but also in the accumulation of secretory fluid in the accessory gland reservoir of males [147]. These findings indicate that the circadian clock is involved in the common mechanism governing photoperiodic diapause of both sexes, but not in sex-specific mechanisms in the reproductive system such as oogenesis shown in *D. melanogaster* [101,102]. Reproductive diapause in insects including *R. pedestris* is induced by suppression of JH secretion from the CA [150-152]. The clock genes in the gut of *P. apterus* are controlled by JH to regulate the local physiological state [120,122]. In *R. pedestris*, however, *per* and *cyc* RNAi also had opposing effects on the expression patterns of JH-repressible and -inducible genes [130]. These results suggest that JH secretion was induced by *per* RNAi but was suppressed by *cyc* RNAi. Moreover, JH analogue application induced ovarian development in *cyc* RNAi insects, i.e., it cancelled the effect of *cyc* RNAi [130]. Based on these results, Ikeno et al. [130] concluded that the circadian clock in *R. pedestris* is involved in an upstream event of the endocrine regulation, such as the photoperiodic clock in the brain.

Thus, the role of clock genes has been confirmed in photoperiodism of some insects, although different interpretations have been made depending on the species and authors. There are two predominant hypotheses: One is that clock genes function in photoperiodism as components of the circadian clock and that the results provide the molecular basis of Bünning’s hypothesis [129,130,147,148]. The other emphasizes the non-clock pleiotropic functions of clock genes [153,154]. Bradshaw and Holzapfel [155] pointed out that the results of Ikeno et al. [130] in *R. pedestris* can also be explained by pleiotropic effects. However, additional experimental results in *R. pedestris* showed that the first hypothesis appears more likely, at least in this species [129,147-149]. A recent study on the photoperiodic regulation of chill-coma recovery times in *D. melanogaster* also supports the first hypothesis [142]. The next question is whether this hypothesis is widely applicable for other insects.

### Circatidal clock

In *A. asahinai*, a PRC of the circatidal rhythm showed the existence of a circatidal clock different from circadian clocks [45]. However, it is also plausible that the circatidal clock evolved from a circadian clock with known clock genes, which is generally present in insects. *A. asahinai* shows both circatidal and circadian rhythms simultaneously in its locomotor activity. After RNAi of a clock gene, *per* or *Clk*, the circadian modulation of activity was cancelled, but the circatidal rhythm persisted with no remarkable changes in its free-running period. Thus, both a negative element of *per* and a positive element of *Clk* in the circadian clock play no role, or a less-important role in the circatidal rhythm. It can therefore be concluded that the circatidal rhythm in *A. asahinai* is controlled by a circatidal clock whose molecular mechanism is different from that of the circadian clock [26,27]. Also in the intertidal crustacean *Eurydice pulchra* (Isopod), RNAi of *per* disrupted the circadian, but not the circatidal, rhythm in its swimming behavior [156].

### Circalunar and circasemilunar clocks

The molecular mechanisms of circalunar and circasemilunar rhythms remain unclear. Different populations of *C. marinus* have different local adaptations in their lunar and diurnal rhythms [16,60]. Kaiser and Heckel [61] constructed a linkage map of the *C. marinus* genome to examine the genetic basis of population variation in clock properties. Quantitative trait locus (QTL) analysis identified two QTLs for lunar emergence time. Mapping of clock and photoreceptor genes identified *ciliary opsin 2* and *cry-d* as candidate genes involved in lunar timing. Although both are photoreceptor genes, Kaiser and Heckel [61] regarded this study as a step toward unraveling the molecular mechanisms of the lunar clock of *C. marinus*.

The marine ragworm *Platynereis dumerilii* (Annelid, Polychaeta) has a circadian clock composed of the same clock genes as those in insects, and a circalunar clock for reproductive cycles. When the oscillation of the circadian clock was disrupted by an inhibitor of casein kinase 1β/ε, which plays a role in the circadian clock by phosphorylating PER, the monthly reproductive cycles continued [157]. Zantke et al. [157] concluded that the circalunar clock in this species is independent of the oscillations of the circadian transcriptional clock. Such an approach may be effective in insects as well.

### Circannual clock

Although no information has been obtained on genes constructing the circannual clock in any organism, Matrai et al. [158] pointed out the possibility that feedback loops consisting of positive and negative elements, similar to those in circadian clocks, are involved in generating the circannual rhythm of marine dinoflagellates. In *A. verbasci*, we suggest that the circadian clock for photoperiodism is related to the circannual clock because Nanda-Hamner experiments showed that a circadian clock is involved in photoperiodic time-measurement for phase resetting of this circannual clock [127]. However, there is no correlation between the pupation time determined by the circannual rhythm and the period of circadian rhythms [22,159]. It is therefore unlikely that the circannual clock of *A. verbasci* shares the same feedback loops of clock genes with the circadian clock. Identification of the molecules involved in this circannual clock is now awaited.

## Conclusions

Although the purpose of this review is to extract common features among diverse insect clocks, the available knowledge differs greatly among these clocks. The circadian clock in the brain that controls daily rhythmic events is best understood. Therefore, we have no choice but to compare other clocks with the brain circadian clock.

PRCs in insect clocks reported to date exhibit close similarities. Such features are not restricted to biological clocks related to environmental cycles, but may be common to many biological oscillators [49]. In fact, Type 1 and 0 phase resetting, and singular behavior have also been shown in some ultradian rhythms, e.g., glycolytic oscillation in yeast [160], and cardiac and neural pacemakers in animals [161-164]. Nevertheless, it is notable that the circannual clock of *A. verbasci* shows a striking similarity in the phase response with the circadian clock for the eclosion rhythm in *D. pseudoobscura* [41,42,46,47]. Hence, we suggest that the circannual clock is a similar physiological mechanism to the circadian clock, even though it has not been clarified at the molecular or neuronal level. Some researchers have assumed in the limit cycle model that state variables of the circadian clock are the levels of components of the molecular feedback loops [165-167]. State variables of the circannual clock of *A. verbasci* may also somehow form a circannual feedback loop at the physiological, biochemical, or molecular level [47].

Insect clocks are classified into central and peripheral clocks based on their anatomical locations. Among central clocks, the optic-lobe circadian clock in cockroaches and crickets and the mid-brain circadian clock in moths and flies are best understood. However, there is no direct evidence that these circadian clocks are involved in rhythmic phenomena other than circadian rhythms in daily events. Peripheral circadian clocks have also been shown to regulate the circadian rhythm in the function of the tissue where they reside [40]. The crucial role of the antennal circadian clock in celestial navigation has been reported in *D. plexippus* [84,85], and Bajgar et al. [120] recently showed that clock genes expressed in the gut are involved in the photoperiodism of the gut status in *P. apterus*. The role of peripheral circadian clocks in various rhythmic phenomena should be examined in the future.

Although many clock genes have been identified by their roles in circadian rhythms in daily events, it is likely that these clock genes are also involved in time-compensated celestial navigation [55,84]. Moreover, recent experimental results show that some of these genes are involved in photoperiodism [129,130,142,145,147,148]. Therefore, the role of clock genes in rhythms other than circadian rhythms in daily events is in the spotlight. Both time-compensated celestial navigation and photoperiodism are physiological mechanisms directly related to a circadian clock. Even if this clock is not the same as the circadian clock for daily events, it has a free-running period of approximately one day and entrains to daily light–dark cycles under natural conditions. It is therefore not surprising that the same molecular network is used for them. In contrast, there is no experimental evidence that any known clock gene is involved in biological clocks with periods different from “approximately one day”. In fact, silencing of clock genes has no effect on the circatidal clock in *A. asahinai* [26,27].

To understand diverse insect clocks more completely, it will be necessary to obtain knowledge to the same level as that accumulated in the central circadian clock that controls daily rhythmic events.

## Abbreviations

CA: Corpus allatum
*Clk*: *Clock*
*cry*: *Cryptochrome*
*cyc*: *Cycle*
JH: Juvenile hormone
LD: Light/dark
*npd*: *Non-photoperiodic-diapause*
PDF: Pigment-dispersing factor
*per*: *Period*
PI: Pars intercerebralis
PL: Pars lateralis
PRC: Phase response curve
QTL: Quantitative trait locus
RNAi: RNA interference
*tim*: *Timeless*
φ_i_: Photo-inducible phase
